# Elucidating White Matter Contributions to the Cognitive Architecture of Affective Prosody Recognition: Evidence from Right Hemisphere Stroke

**DOI:** 10.3390/brainsci15070769

**Published:** 2025-07-19

**Authors:** Meyra S. Jackson, Yuto Uchida, Shannon M. Sheppard, Kenichi Oishi, Ciprian Crainiceanu, Argye E. Hillis, Alexandra Z. Durfee

**Affiliations:** 1 Department of Neurology, Johns Hopkins School of Medicine, 600 N Wolfe Street, Baltimore, MD 21287, USAargye@jhmi.edu (A.E.H.); 2 School of Psychology, University of Sussex, Falmer, Brighton BN1 9RH, UK; 3 Department of Radiology, Johns Hopkins School of Medicine, 111 Market Place, Baltimore, MD 21202, USA; yuchida2@jhmi.edu (Y.U.);; 4 Department of Biostatistics, Johns Hopkins University, 615 N Wolfe Street, Baltimore, MD 21205, USA; 5 Department of Physical Medicine and Rehabilitation, Johns Hopkins School of Medicine, 600 N Wolfe Street, Baltimore, MD 21287, USA; 6 Department of Cognitive Sciences, Johns Hopkins University, 3400 N Charles Street, Baltimore, MD 21218, USA; 7 Department of Speech-Language Pathology & Audiology, Towson University, 8000 York Road, Towson, MD 21252, USA

**Keywords:** emotion, prosody recognition, receptive aprosodia, right hemisphere, stroke recovery, white matter

## Abstract

**Background/Objectives**: Successful discourse relies not only on linguistic but also on prosodic information. Difficulty recognizing emotion conveyed through prosody (receptive affective aprosodia) following right hemisphere stroke (RHS) significantly disrupts communication participation and personal relationships. Growing evidence suggests that damage to white matter in addition to gray matter structures impairs affective prosody recognition. The current study investigates lesion–symptom associations in receptive affective aprosodia during RHS recovery by assessing whether disruptions in distinct white matter structures impact different underlying affective prosody recognition skills. **Methods**: Twenty-eight adults with RHS underwent neuroimaging and behavioral testing at acute, subacute, and chronic timepoints. Fifty-seven healthy matched controls completed the same behavioral testing, which comprised tasks targeting affective prosody recognition and underlying perceptual, cognitive, and linguistic skills. Linear mixed-effects models and multivariable linear regression were used to assess behavioral performance recovery and lesion–symptom associations. **Results**: Controls outperformed RHS participants on behavioral tasks earlier in recovery, and RHS participants’ affective prosody recognition significantly improved from acute to chronic testing. Affective prosody and emotional facial expression recognition were affected by external capsule and inferior fronto-occipital fasciculus lesions while sagittal stratum lesions impacted prosodic feature recognition. Accessing semantic representations of emotions implicated the superior longitudinal fasciculus. **Conclusions**: These findings replicate previously observed associations between right white matter tracts and affective prosody recognition and further identify lesion–symptom associations of underlying prosodic recognition skills throughout recovery. Investigation into prosody’s behavioral components and how they are affected by injury can help further intervention development and planning.

## 1. Introduction

The focus of discourse is often on linguistic information, and rightfully so. A great deal of information is conveyed in those combinations of words, phrases, and sentences to express thoughts, intents, emotions, and desires. However, non-verbal information, including facial expressions (extralinguistic) and prosodic (paralinguistic) emotion, is conveyed in not only *what* we say but also *how* we say it. Modulation of acoustic features in one’s voice can give rise to emotional meaning, termed affective (emotional) prosody [1]. Happiness, for example, is typically characterized by a faster speaking rate, greater variability in fundamental frequency (F0), and higher intensity, resulting in both energetic and melodic sound [2]. Sadness, on the other hand, is conveyed by a slower speaking rate, lower intensity, and higher spectral noise, creating the effect of a broken voice [2]. Prosody impairments, termed aprosodia, can be expressive and/or receptive in nature, and though expressive aprosodia may be more evident than receptive affective aprosodia to observers, receptive affective prosody deficits can be just as harmful to discourse success [3,4,5,6] and thus serve as the focus of the current investigation.

Receptive affective aprosodia, or difficulty in the recognition of acoustic features, such as pitch, rate, loudness, and rhythm, associated with the expression of a given emotion, is a common sequela of right hemisphere stroke (RHS) [7,8,9,10,11]. Estimates indicate that receptive affective aprosodia can occur in up to 70% of patients with RHS during the acute recovery stage, persisting in 12–44% of patients across subacute and chronic stages [8]. As suggested in Dara and colleagues’ work [7], prosody recognition deficits might be an even finer marker of stroke outcome than neglect or extinction, which are typically listed as hallmark RHS symptoms. Prosodic deficits were reported as a top concern by nearly a third of caregivers of RHS patients [4] and likely contribute to reduced social networks in this clinical population [6,12] due to communication breakdowns and misunderstandings between interlocutors [13,14]. However, after RHS, prosody is infrequently identified, rarely assessed, or at best assessed subjectively, as post-RHS recovery commonly focuses on other skills and functions, such as cognitive functions (e.g., hemispatial visual neglect/inattention, memory) and motor skills [4,15]. While the rehabilitation of mobility and activities of daily living is important for working towards independence, it is oftentimes not sufficient for patients and caregivers [4], especially in cases where communication is affected by stroke.

The last thirty-five years have yielded theoretical models that include neuroanatomical correlates and a cognitive architecture underlying prosody. Schirmer and Kotz [2] propose their three-stage Working Model for the Processing of Emotional Prosody. Stage 1, sensory processing, involves the analysis of acoustic features of speech processed in both hemispheres of the brain—mainly within auditory cortices and their projections to the superior temporal sulcus [16]. In Stage 2, the integration stage, emotionally meaningful acoustic cues are encoded along the “what” pathway, which originates in the superior temporal gyrus and projects to the anterior superior temporal sulcus, lateralized to the right hemisphere. Lastly, in Stage 3, the cognition stage, higher-order cognitive processes, such as evaluative judgements of emotionally meaningful information, are associated with activity in the right inferior frontal gyrus and orbitofrontal cortex. Stage 3 also comprises the integration of emotional prosody with language in the left inferior frontal gyrus [16,17], highlighting the complex interhemispheric processing needed for accurate prosodic decoding.

**Figure 1:**
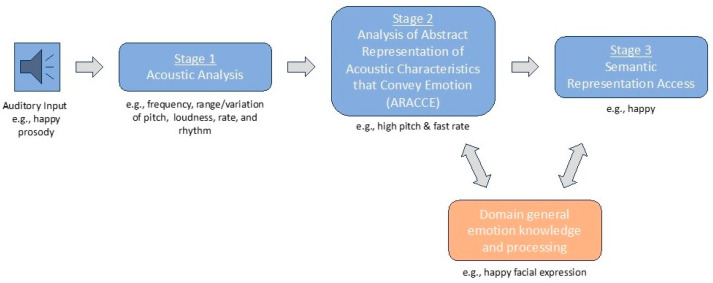
Proposed cognitive architecture of affective prosody recognition.

Other models describe a similar model to Schirmer and Kotz [2], with additional details paralleling propositional linguistic processing, elaborating on the underlying cognitive–linguistic processes engaged, and providing empirical evidence for distinct impairment loci and subtypes of affective aprosodia [18,19,20] (see Figure 1). Stage 1 comprises analysis of acoustic features of speech, such as differences in pitch, loudness, rhythm, and rate. During Stage 2, the abstract representation of acoustic characteristics that convey emotion (ARACCE) is accessed, and prosodic features are matched to an emotion, creating an emotional prosody profile of defining prosodic characteristics. ARACCE can be compared to an emotional prosody lexicon that is typically shared among people speaking the same language and culture (e.g., happy prosody in English: fast rate, high pitch, loud volume). In Stage 3, the semantic representation of an emotion is accessed and retrieved, allowing a certain emotion to be interpreted. As further refined by Sheppard and colleagues [18], Stages 2 and 3 interact with domain-general processes of emotion, such as via facial expressions [20].

Though receptive affective aprosodia has been investigated for decades, understanding the lesion correlates of its underlying cognitive architecture is in its relative infancy. Seminal work by Ross [21] observed varying aprosodia symptoms depending on lesion localization, mirroring the classical locationist aphasia classification system (e.g., motor aprosodia, transcortical sensory aprosodia) [22]. Though these subtypes were critical in demonstrating that aprosodia is not a homogenous post-stroke symptom by identifying patients with unique behavioral clusters, this subtyping does not as strongly explain why prosodic recognition impairments occur [14]; that is, what are the deficient underlying perceptual, cognitive, and linguistic skills that are result in receptive affective aprosodia? Answers to this question are critical for clinicians, primarily speech-language pathologists (speech therapists), to conduct sound assessment and to plan targeted, effective interventions for individuals post-RHS demonstrating receptive affective aprosodia, for which there is a dearth of evidence-based resources available. Additionally, early evidence suggests a benefit to targeting underlying prosody recognition skills to improve overall affective prosody recognition abilities [23].

A growing evidence base elucidates the collaborative bihemispheric involvement in prosody processing [16,17,24,25,26,27], but data from clinical [10,19,22,25,28,29,30,31] and healthy adult [16,17,24,26,27,32,33,34] populations implicate the right hemisphere as playing a critical, and arguably central, role in affective prosody recognition. Right temporoparietal regions appear attuned for spectral analysis and better process slower time-scale acoustic signal changes, requiring longer integration times compared to left homologues that process and integrate temporal and linguistic information [8,17,19,32,35]. Moreover, an increasing body of evidence in both healthy and clinical populations supports a dual-stream organization of prosody in the right hemisphere [24,36,37,38,39] that mirrors the dual stream model of propositional language processing in the left hemisphere [35,40]. According to a recent systematic review of aprosodia following RHS [36], receptive affective prosody deficits seem to result from damage to more ventrally located structures, while dorsally situated structures appear to be more often associated with expressive affective prosody deficits. However, other studies have reported dorsal and ventral structures associated with receptive [37] and expressive prosody [41], respectively, highlighting that the aforementioned dual-stream division of labor for prosodic processing is not mutually exclusive.

**Figure 2:**
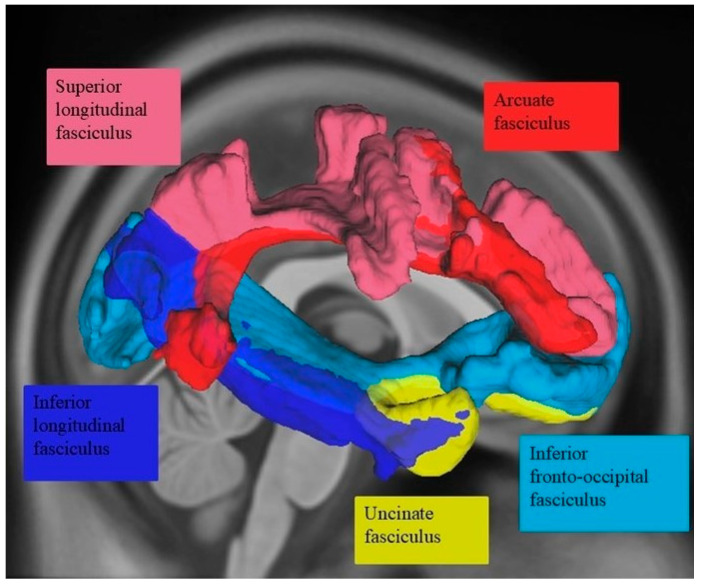
Right hemisphere white matter tracts previously implicated in affective prosody recognition.

The neural substrates of prosody recognition include not only gray matter but also white matter structures in the right hemisphere [28,38,39,42], such as the inferior longitudinal fasciculus, inferior frontal occipital fasciculus, and extreme capsule (see Figure 2). A diffusion tensor imaging study [38] found that poor affective prosody perception was associated with poorer integrity (i.e., reduced volume and/or lower fractional anisotropy) of the right uncinate and inferior fronto-occipital fasciculi as well as the corpus callosum. The authors found no consistent involvement of left hemisphere structures during affective prosody perception. Additionally, Durfee and colleagues [23] observed that greater damage to the inferior fronto-occipital and inferior longitudinal fasciculi was associated with smaller gains in affective prosody recognition accuracy following a brief training. The arcuate fasciculus appears to play a role in linguistic prosody recognition, specifically as part of the action–perception network, facilitating auditory feedback control as well as aiding in higher-level cognitive integration steps of identifying and explicitly labeling the prosodic contour heard [37]. Grandjean [28] also notes this same labeling process and the connectivity between the superior temporal sulcus/gyrus and inferior frontal gyrus for affective prosody but does not name the connecting structure. Thus, disruption of the white matter pathways connecting gray matter hubs involved in prosodic decoding can contribute to the receptive affective aprosodia symptoms observed post-RHS.

While complementary multi-stage cognitive architecture models [2,18,20,28] and the dual-stream neuroanatomical model for receptive (and expressive) affective prosody [29,36] have established themselves as working models in prosody research, the role of white matter structures specifically in support of these underlying emotional, cognitive, and linguistic processes remains unclear. Previous work has oftentimes focused on gray matter structures and included only a few white matter structures, such as the arcuate fasciculus or sagittal stratum [8,18,38]. Therefore, this study investigated the involvement of right white matter pathway integrity, assessed via lesion volume, fractional anisotropy, and mean diffusivity, in affective aprosodia recovery to better understand network integrity. This study partially replicates lesion–symptom mapping work in affective prosody recognition [38] while extending on previous research by evaluating the role of right hemisphere white matter tracts within the context of the three-stage cognitive architecture models [18]. Several hypotheses regarding the effect of damage to white matter structures in relation to underlying receptive affective prosody processes were tested:

1. According to the dual-stream model of prosody processing [36], damage to right hemisphere ventral stream white matter structures, such as the inferior fronto-occipital fasciculus and uncinate fasciculus, is expected to be more frequently associated with impairments in affective prosody recognition. In contrast, damage to more dorsally situated white matter pathways, such as the arcuate fasciculus as captured within the superior longitudinal fasciculus, would not be as commonly associated with affective prosody recognition deficits.
2. Damage within specific ventral stream white matter structures is also expected to impact specific stages underlying prosody recognition. According to Schirmer and Kotz’s model [2], more posterior right hemisphere white matter structures (e.g., sagittal stratum) would be associated with earlier stages of prosody recognition, whereas damage to more anterior structures (e.g., uncinate fasciculus) would likely affect later stages of prosody recognition.

## 2. Materials and Methods

### 2.1. Participants

The study was conducted in accordance with the Declaration of Helsinki and approved by the Institutional Review Board of Johns Hopkins University (NA_00042097). The initial approval date was 21 October 2010, and the protocol and results were reviewed and approved yearly since that date. The latest approval date was 11 July 2024. Recruitment began after 2011. Adults with acute RHS were recruited upon their inpatient admittance to Johns Hopkins Hospital as part of an ongoing longitudinal study of RHS recovery. Study team personnel screened admitted patients and approached eligible participants and their families within five days of stroke symptom onset to obtain informed consent. Informed consent was obtained from all subjects or their legally authorized representatives involved in the study. Inclusion criteria encompassed RHS, normal/corrected-to-normal vision and hearing (determined via participant report, interview, and medical record review), and 18 years of age or older. Exclusion criteria included any type of other significant neurological or psychiatric history, primary hemorrhagic stroke, participants whose acute stroke extended beyond the right hemisphere, and history of previous symptomatic stroke. Handedness, as determined via chart review and/or interview, was not exclusionary; rather, if a participant reported left-handedness, a confrontation naming task was administered (Boston Naming Test, Second Edition [43]) and a language sample elicited (picture description [44,45]) to assess for characteristics of aphasia. If aphasia was suspected, prosody assessment was terminated.

Behavioral testing and structural magnetic resonance imaging (MRI) were completed within seven days of symptom onset (acute) and then again at 2–4 months (subacute), 5–7 months (early chronic), and 11–13 months (late chronic) post-stroke.

Participants were assessed for presence of hemispatial visual neglect/inattention via a modified circle-gap detection task [46] or visual scene copy. If inattention/neglect was suspected, prosody testing procedures were modified (e.g., stimuli placement, participant cueing to turn head) to maximize visual attention. During experimental tasks, research personnel checked whether participants could see the screen fully by asking participants to read aloud/point to named stimuli.

To validate the behavioral tasks utilized with post-RHS participants, a control group of age- and education-matched healthy adults over 18 years of age and with no reported vision and hearing impairments nor history of neurological disease or damage were also recruited to complete the same prosody and emotion tasks as completed by participants with RHS. Controls’ accuracy on behavioral tasks was used to determine cutoffs for impairment for participants with RHS.

### 2.2. Procedures: Behavioral Testing

**Figure 3:**
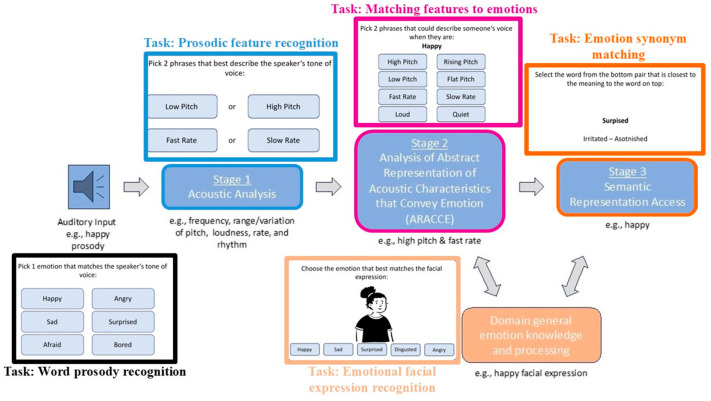
Graphical depiction of how each behavioral task addresses different stages of the proposed affective prosody recognition cognitive architecture.

A battery of emotional and prosodic tasks was completed by participants, and the receptive tasks are briefly reported here. For an in-depth description of the following tasks, please refer to Sheppard and colleagues [18]. See Figure 3 for an example of each task and the stage of the cognitive architecture model that each task was intended to target. The tasks were administered by a speech-language pathologist or a research assistant trained by a speech-language pathologist in the execution and scoring of these tasks. Participants were tested in their hospital room (acute timepoint) or in a quiet outpatient clinic room (subacute and chronic timepoints). The tasks as detailed below were presented via a PowerPoint presentation on a laptop computer (see https://score.jhmi.edu/downloads.html (accessed on 20 December 2024) for stimuli), with audio presented via laptop computer speakers. Presentation of task multiple-choice options was oriented centrally on the laptop computer screen unless otherwise specified. Task instructions were read aloud by the test administrator and presented visually to participants. Participants could respond verbally or gesturally. Across behavioral tasks, impairment was defined as a score at or below the fifth percentile of control participants’ scores on the same task. Testing at the acute timepoint occurred either in a single or across multiple sessions, with testing completed within the acute time frame defined for this study (i.e., within seven days of stroke symptom onset). Testing occurred in a single session at subacute and chronic testing timepoints.

#### 2.2.1. Affective Prosody Recognition (i.e., Word Prosody Recognition)

Recordings of 24 semantically neutral sentences spoken with affective prosody (i.e., happy, sad, angry, afraid, surprised, bored) were presented to participants. Participants were then asked to decide which emotion the speaker was conveying through their tone of voice. For participants with RHS, a score at or below the cutoff on this task was considered evidence of receptive affective aprosodia. No feedback was provided.

#### 2.2.2. Recognition of Prosodic Features in Speech (Stage 1)

Participants listened to the same 24 sentences presented during the affective prosody recognition task and were asked to identify key prosodic features in a two-option forced choice format (i.e., Was the rate fast or slow? Was the volume loud or quiet? Was the pitch high or low?). This task assessed Stage 1 of affective prosody processing by applying acoustic terms introduced during a pure tone identification not discussed here (cf. Sheppard et al., 2021 [18]) to prosodic stimuli. Feedback regarding accuracy as well as the correct response were provided after each of the 24 trials as this task served as part of a training battery for prosody recognition [23].

#### 2.2.3. Matching Features with Emotions (Stage 2)

Participants were presented with the same emotions targeted in previously mentioned prosody tasks and were asked to select two to three prosodic features that best described how to express a specific emotion through one’s voice, resulting in six trials (one trial per emotion). Prosodic feature profiles for each emotion were identical to those identified during the Stage 1 task and were determined by previous research on common prosodic features of emotions [47]. This task targeted Stage 2 of affective prosody processing or ARACCE access. Feedback regarding accuracy as well as the correct response were provided after each trial as this task served as part of a training battery for prosody recognition.

#### 2.2.4. Emotion Synonym Task (Stage 3)

To assess Stage 3 of affective prosody recognition (emotion semantic representation access), participants were presented with a target emotion (e.g., happy) at the top of the laptop computer screen and were asked to choose which of two options (e.g., cheerful or helpless) presented below the target emotion was closest in meaning to the emotion. There were 24 trials total, with 4 trials each for happiness, sadness, anger, fear, surprise, and disgust. No feedback was provided.

#### 2.2.5. Facial Expression Recognition

To assess emotion recognition abilities in other modalities, participants completed an emotional facial expression recognition task. Pictures of people expressing different emotions via facial expressions were presented centrally on a laptop computer screen. Five emotions—happiness, sadness, anger, disgust, and surprise—were presented below the pictures in a single row in the same order across trials. There were eight trials of each emotion, resulting in 40 trials total. Participants selected the emotion that best matched the emotion expressed in the picture. No feedback was provided.

### 2.3. Image Acquisition and Processing

Using a 3.0T Siemens Trio scanner (Siemens, Washington, DC, USA) located at Kennedy Krieger Institute or Johns Hopkins Hospital, multiple research MRI sequences were acquired at each of the timepoints (acute, subacute, early chronic, late chronic), including diffusion weighted imaging (DWI) and diffusion tensor imaging (DTI) sequences, to visualize acute lesions and to evaluate structural changes and differences in white matter connectivity in white matter structures of interest, respectively. Since few participants completed research MRI scans acutely, clinical DWI scans were included in the study from 1.5T or 3.0T Siemens scanners. Acute clinical DWI scans were traced manually using MRIcron by trained technicians (authors MSJ and AZD) and supervised by a neurologist (author AEH). Tracings were normalized to MNI space using SPM12 (https://www.fil.ion.ucl.ac.uk/spm/software/spm12/ accessed on 20 December 2024). Normalization was completed on DWI images with a healthy older adult template [48], with warping parameters applied to lesion traces. Percent damage to regions of interest (ROIs) was calculated for each participant using the JHU atlas [49]. There are several methods to quantify scalar values derived from DTI. These methods can generally be divided into two categories: atlas-based and tract-based quantifications. The tract-based approach, which includes techniques based on tractography, focuses on analyzing the connections between two distinct regions. In contrast, the atlas-based approach excels at evaluating tracts within their specific locations. In this study, the atlas-based approach was utilized to identify the locations of stroke-related alterations in white matter.

DTI sequences (voxel size: 0.83 × 0.83 × 2.00 mm, TR: 6998 ms, TE: 75 ms, acquisition matrix: 96 × 96, # axial slices: 70, slice thickness: 2.20 mm, b-value: 0 and 700 s/mm^2^, diffusion gradient directions: 34, number of excitations: 2) were linearly registered to the b0 image, followed by voxel-wise tensor fitting using DTI Studio (www.mristudio.org, accessed on 20 December 2024) [50]. The fractional anisotropy (FA) map was calculated from the tensor field. The JHU-DTI multi-atlas was applied to each participant’s FA map, creating the parcellation map that contains 168 anatomical areas as the ROIs [49]. Then, the FA values of the 168 ROIs were extracted. DTI scans were available at acute (*n* = 6), subacute (*n* = 12), early chronic (*n* = 10), and late chronic (*n* = 13) timepoints.

Based on previous findings [22,23,36,37,38], several right hemisphere white matter tracts were considered as possible ROIs in subsequent analyses: inferior fronto-occipital fasciculus, external capsule, internal capsule, sagittal stratum, superior longitudinal fasciculus, inferior longitudinal fasciculus, uncinate fasciculus, and corpus callosum (body, genu, tapetum). Of these identified white matter ROIs, a subset was included in subsequent analyses if the ROI sustained at least 2% damage from the RHS and at least 25% of the participant sample demonstrated damage to that region.

### 2.4. Statistical Analyses

Due to the small sample size, the early and late chronic timepoints were collapsed into a singular chronic timepoint, and data from participants’ latest chronic follow-up visit were included in analyses. To determine whether there was a significant difference in performance in prosody recognition and its subprocesses for the patient group, performance between healthy controls and participants with RHS were compared using linear mixed effects models. Time (acute, subacute, chronic) and Group (participants with RHS, controls) were included as independent variables. Participants were included as random intercepts. Percent accuracy on the behavioral tasks were included as dependent variables, resulting in five separate models (one model per task). If a significant effect of Group was observed, follow-up tests controlling for multiple comparisons using Dunnett’s method were conducted to assess control and RHS group performances at each timepoint (control vs. RHS acute, control vs. RHS subacute, control vs. RHS chronic). If a significant effect of time was observed, follow-up tests controlling for multiple comparisons using Tukey’s HSD were conducted to assess RHS group performance over time (acute vs. subacute vs. chronic).

To determine the white matter structures implicated in prosody recognition and underlying recognition stages, multivariable linear regression was used to assess neural correlates of acute prosody performance. For the regression models, demographic variables (i.e., age, education), acute lesion volume, and percentage damage to white matter ROIs were entered to assess their associations with percent accuracy on behavioral tasks. If multiple ROIs were identified, resulting in separate models for each ROI per task, then the Benjamini–Hochberg procedure (False Discovery Rate [FDR]; corrected α = 0.05) was applied to correct for multiple comparisons at the model level. This exploratory statistical approach was chosen due to the number of statistical comparisons planned and the size of the participant sample. Significant ROIs identified via this procedure will be used *a priori* in a follow-up study with a larger participant sample to provide confirmatory evidence of current study findings.

Finally, linear mixed effects models were utilized to assess the association between common DTI metrics of white matter structures and prosody recognition abilities over time. Fractional anisotropy and mean diffusivity values of ROIs identified during lesion–symptom mapping analyses were included as independent variables that interacted with time, and associated behavioral task performance percent accuracy was included as the dependent variable. Participants were included as random intercepts. Multiple comparisons were corrected using Benjamini–Hochberg procedures (FDR-corrected α = 0.05) as appropriate.

## 3. Results

Twenty-eight participants post RHS (17 men, 11 women; age: M = 57.04 ± 14.42 years; education: M = 15.37 ± 3.53 years; 24 right-handed, 4 left-handed) were included in the sample with a total of 61 observations across all timepoints (24 observations acutely, 14 observations sub-acutely, and 23 observations chronically). A total of 57 healthy adults (N = 57; 34 men, 23 women; age: M = 59.72 ± 13.47 years; education: M = 16.17 ± 2.93 years; 53 right-handed, 4 left-handed) completed varying combinations of the same emotional-prosodic tasks as completed by participants with RHS. Participants with RHS and healthy controls did not differ in age (*t*(83) = 0.86, *p* = 0.40), years of education *t*(82) = 1.37, *p* = 0.18), sex (*X^2^*(1) = 0.01, *p* = 0.92), or handedness (Fisher’s Exact *p* = 0.43). Reported race differed between groups (Fisher’s Exact *p* = 0.001; [participants with RHS–Controls], White/Caucasian [16–42], Black/African America [12–4], Asian [0–2]).

Overall performance accuracy was assessed per timepoint and was variable across time and task (e.g., performance improvement, decline, and plateau observed over one year; see Table 1). Table 1 also lists the normative values for the mean accuracy and standard deviation (SD) for task performance across time for RHS participants as well as healthy controls. Table 2 includes the cut-off scores that determined whether a participant would be classified as impaired on a task as well as the number of patients in our sample that were deemed impaired per task per timepoint.

### 3.1. Behavioral Assessment Results: Between-Group Comparisons

Linear mixed effects models investigating behavioral performance (percent accuracy) on experimental tasks indicated a significant effect of Group (controls > participants with RHS; all *p* < 0.05), but this main effect of Group consistently interacted with Time. For affective prosody and facial expression recognition, controls were more accurate than participants with RHS acutely (word prosody recognition: *t*(90.8) = −4.24, *p* < 0.001; facial expression recognition: *t*(80.8) = −2.79, *p* = 0.018). Controls were also more accurate than participants with RHS on word prosody recognition subacutely (*t*(101.8) = −2.43, *p* = 0.046). For recognition of prosodic features (Stage 1 task), controls were more accurate than participants with RHS at acute testing only (*t*(95.3) = −3.32, *p* = 0.004). For emotion synonym matching (Stage 3), controls were more accurate than participants with RHS at all timepoints (acute: *t*(82.2) = −3.63, *p* = 0.001; subacute: *t*(88) = −3.57, *p* = 0.002; chronic: *t*(81.3) = −2.50, *p* = 0.040). All other contrasts were not statistically significant (*p* > 0.05). To summarize, on all tasks outside of Matching Features with Emotions, healthy controls performed significantly better than patients at the acute timepoint. Controls demonstrated significantly higher accuracy on Word prosody recognition subacutely as well. Finally, in Emotion Synonym Matching, healthy controls performed better than RHS participants across all timepoints.

### 3.2. Behavioral Assessment Results: Within-Group (RHS) Comparisons

**Figure 4:**
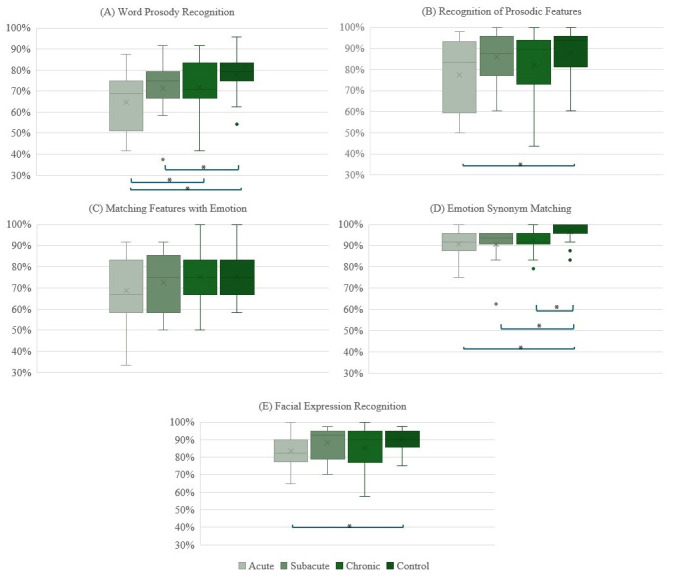
Box-and-whisker plots for RHS participants’ performance (% accuracy) across timepoints as well as healthy matched control participants’ performance on the various tasks (* *p* < 0.05). Dots below box-and-whisker plots in parts (**A**,**D**) indicate outlier performance.

Time, specifically chronic vs. acute, performance on Word Prosody Recognition was observed (Β = 7.4, *p* = 0.003). Patients with RHS were more accurate at chronic compared to acute testing in recognizing affective prosody (Word prosody recognition; *t*(37.3) = −3.12, *p* = 0.010). All other within-group comparisons were non-significant (*p* > 0.05) (see Figure 4).

### 3.3. Lesion–Symptom Mapping Results

**Figure 5:**
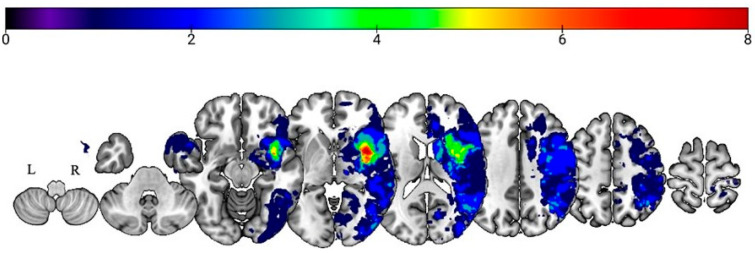
Lesion overlays indicating the number of RHS participants with overlapping lesions (neurological view).

Through the inspection of acute lesion overlay maps (Figure 5), the external capsule (EC), insula, posterior insula, inferior fronto-occipital fasciculus (IFOF), putamen, and uncinate fasciculus (UF) demonstrated the greatest lesion overlap in the participant sample. Using the inclusion criteria as described above to identify ROIs, seven right hemisphere white matter structures were included in subsequent analyses: IFOF, superior longitudinal fasciculus (SLF), UF, EC, sagittal stratum (SS), and body and genu of the corpus callosum (BCC and GCC, respectively).

Robust linear regressions were employed upon examination of model multivariable linear regression residual diagnostics. Thirty-five separate models were run to assess lesion–symptom associations at the acute timepoint (7 ROIs × 5 behavioral tasks = 35 models), so multiple comparison correction procedures (FDR-corrected *α* = 0.05) were utilized at the model level. In the interest of space, significant models will be reported in detail, and non-significant models will be summarized in the body of the manuscript. The full list of nonsignificant models and their respective values can be found in Supplementary Materials (Tables S1–S10).

#### 3.3.1. Prosody Recognition

For acute prosody recognition performance, a negative association with damage to the EC and IFOF was found—lower accuracy was associated with greater damage to these structures (*p* = 0.032 and *p* = 0.0027, respectively). Percent damage to the SLF and prosody recognition accuracy showed a positive association (*p* = 0.025; see Table 3).

#### 3.3.2. Recognition of Prosodic Features in Speech (Stage 1) Findings

As for recognition of prosodic features in speech, only damage to the SS was found to have a negative association with recognition of prosodic feature task accuracy, indicating lower accuracy with greater damage to the SS (*p* < 0.001). Damage to the SLF (*p* = 0.046), UF (*p* = 0.002), BCC (*p* = 0.006), and GCC (*p* = 0.004) were positively associated with prosodic feature recognition accuracy (see Table 4).

#### 3.3.3. Matching Features with Emotions (Stage 2) Findings

Only a positive association between damage to the SLF and matching features with emotions accuracy was found (*p* = 0.001; see Table 5).

#### 3.3.4. Emotion Synonym Matching (Stage 3) Findings

A negative association was found between performance on emotion synonym matching and damage to the SLF (*p* = 0.004). Worse performance was associated with greater SLF damage. Both the BCC (*p* < 0.001) and GCC (*p* < 0.001) percent damage demonstrated a positive association with this task (see Table 6).

#### 3.3.5. Facial Expression Recognition (Domain-General Emotion Processing)

Like word prosody recognition accuracy (i.e., affective prosody recognition), facial expression recognition accuracy was negatively associated with percent damage to the EC (*p* = 0.021) and IFOF (*p* = 0.007). Thus, greater damage to these white matter structures in the right hemisphere corresponded to worse performance on facial expression recognition (see Table 7).

### 3.4. Association of DTI Metrics with Affective Prosody Recognition Across Recovery

Mean diffusivity and fractional anisotropy values from ROIs identified as significant during lesion–symptom mapping (robust regression) analyses were entered as independent variables interacting with time to assess associations with corresponding task performance. More specifically, we explored the association between (1) IFOF and EC fractional anisotropy and mean diffusivity values with prosody recognition and facial expression accuracy, (2) SLF fractional anisotropy and mean diffusivity with emotion synonym accuracy, and (3) SS fractional anisotropy and mean diffusivity with recognition of prosodic feature accuracy across acute, subacute, and chronic timepoints, resulting in 12 separate models (2 DTI metrics × 6 lesion–symptom pairs). Significant associations among SLF mean diffusivity values and emotion synonym matching performance at acute (*p* = 0.02), subacute (*p* = 0.03), and chronic (*p* = 0.03) timepoints were observed (*X*^2^(3) = 8.45, *p* < 0.04). However, the model did not remain significant following FDR correction (corrected model *p* = 0.075). No other significant associations were observed among DTI metrics and behavioral task accuracy.

## 4. Discussion

The right hemisphere has been described as housing a “non-verbal affect lexicon”, which includes representations of prosodic contours associated with varying emotions [18,20]. As Wright and colleagues [19] demonstrated with case studies, damage to certain right hemisphere structures could result in a loss of access to various aspects of this lexicon. If this supposition is made specifically to right hemisphere white matter structures, then disruption of key connections among gray matter affective prosody recognition hubs would also result in a loss of access to or transfer of relevant affective prosodic information within the prosody processing network. Indeed, previous work has implicated disruptions in right hemisphere white matter structures with impaired affective prosody recognition and amusia [38], but the authors did not investigate associated underlying prosodic skills and white matter tract integrity. The literature on neurological conditions impacting white matter integrity, such as multiple sclerosis, has also observed affective prosody recognition deficits [51,52], further highlighting the role of structural (dis)connectivity in affective prosody recognition abilities. Thus, the current work extends the literature by highlighting that damage to specific white matter pathways disrupts distinct processes proposed to be foundational to affective prosodic recognition and that recovery of these skills varies over the first year after stroke.

When comparing task performance between participant groups, significant differences were observed primarily at the acute stage. Specifically, participants with RHS demonstrated significantly lower accuracy during affective prosody recognition as well as posited prosody-supporting skills—acoustic–prosodic decoding (Stage 1; Recognition of Prosodic Features in Speech task), semantic representation of emotion access (Stage 3; Emotion Synonym Matching task), and domain-general emotion recognition (Facial Expression Recognition task)—compared to healthy matched controls. Affective prosody recognition continued to demonstrate weaker performance at subacute testing for participants with RHS, but participant groups did not significantly differ on the task at chronic testing. Controls were also significantly more accurate on the Emotion Synonym Matching task than participants with RHS at subacute and chronic testing. These findings, coupled with the significant improvement in affective prosody recognition performance from acute to chronic testing in RHS participants, highlight that receptive affective prosody and underlying skills improve throughout the first year of recovery. There was a relative strength of ARACCE access throughout recovery as performance on matching emotions to prosodic profiles was statistically similar across participant groups. Across all prosody tasks, large variations in performance were observed, which falls in line with findings of prosody recognition variability in healthy adults depending on the emotion expressed [53].

Damage to multiple right hemisphere white matter structures—namely IFOF, EC, and UF—was implicated in poor behavioral task performance, and these structures have previously been associated with receptive affective prosody impairments [28,29,30]. In partial support of our hypotheses, different white matter structures were significantly associated with different prosody and emotion processing tasks. Lesions to the EC and IFOF were associated with impaired performance in emotional prosody recognition as well as facial expression recognition. As a major white matter structure connecting parietal and occipital regions to frontal areas, the IFOF acts as a major information transmission pathway with the potential to influence visual and semantic (including language) processing as well as goal-oriented behavior [54,55]. In addition to subserving important semantic processes, the IFOF also appears integral for processing unique rhythmic and pitch information for speech and music. Sihvonen and colleagues [38] observed that right IFOF integrity was significantly associated with not only affective prosody recognition but also linguistic prosody (e.g., lexical stress, type of sentence—statement vs. command) recognition and musical pitch and rhythm processing at three weeks and three months post-stroke. Thus, disruptions of the IFOF, via stroke for example, could result in the disconnection between early-stage prosody processing areas covering acoustic analysis through later-stage processes, such as the access to semantic representations of emotions, making the IFOF a major player in receptive affective prosody processing.

In addition to the IFOF, the EC was also implicated in domain-general emotion processing as evidenced by its integrity associated with affective prosody and emotional facial expression recognition in the current study. Research by Ethofer and colleagues [56] outlined the EC as the link between right superior temporal sulcus and orbital inferior frontal gyrus, connecting voice-sensitive processing areas. What is more, the EC’s involvement in basal ganglia processing by facilitating frontostriatal projections between caudate and lateral putamen supports its role in more domain-general emotion processing as the basal ganglia is involved in emotion recognition and expression [57] among other skills. Efthymiopoulou and colleagues [58] observed left EC involvement during production of emotional but not non-emotional discourse in participants with left hemisphere stroke, further supporting the white matter tract’s role in emotion processing irrespective of domain.

Lesions to the SS, which includes portions of the ILF, IFOF, and optic radiations [59] and which connects the thalamus to the cortex [60], were found to be associated with worse performance on recognition of prosodic features, an early stage of affective prosody recognition [2,18,28]. The observed effect of the SS on recognition of prosodic features supports our hypothesis and aligns with previous models [2] and dual stream accounts [2,29,36] of prosody recognition: the SS helps to transfer auditory information from sensory processing hubs to higher level cognitive integration in the cortex in support of affective prosody recognition. Work by Davis and colleagues [60] found that the SS integrity independently predicted sarcasm recognition in a group of adults following acute RHS. However, previous work from our group failed to identify an association among affective prosody recognition abilities and SS damage. These discrepant findings appear to be influenced by the aims of previous and current investigations. The first study [8] assessed SS damage associations with affective prosody recognition accuracy, and, like this first study, the current study also found no association with this behavioral task. Rather, we observed an association between SS damage and prosodic feature recognition performance, which was not investigated by Sheppard and colleagues [8]. A follow-up inquiry [18] did investigate neural substrates of prosodic feature recognition but included participants with RHS and receptive affective aprosodia whereas the current study included RHS participants with and without receptive affective aprosodia. Thus, SS damage may not be the most discriminative predictor of prosodic decoding impairment presence or severity (as assessed on the specific task) among individuals with receptive affective aprosodia; rather, SS damage may better differentiate prosodic decoding abilities between aprosodic and non-aprosodic individuals following acute RHS.

For the most part, interpretation of the regression associations for key white matter structures in the right hemisphere—namely IFOF, SLF, EC, SS—were expected; that is, greater white matter damage was associated with lower accuracy, and thus worse performance, on behavioral tasks. Unexpectedly, positive lesion–symptom associations were observed for other white matter ROIs. Greater percent damage to the UF was associated with higher accuracy on prosodic feature recognition, corpus callosal damage (specifically BCC and GCC) was associated with higher accuracy for prosodic feature recognition and emotion synonym matching, and more SLF damage was associated with higher accuracy during affective prosody recognition, prosodic feature recognition, and matching prosodic features to emotions tasks. We are not suggesting from these findings that greater damage advantageously resulted in better performance; rather, we posit that greater damage sustained to these white matter ROIs resulted in less relative impairment on these tasks compared to participants who performed more poorly on the tasks since participants were included in the study based on lesion rather than symptom presence [61].

Moreover, at first glance, the finding that SLF integrity was associated with emotional semantic representation access (Stage 3) appeared to refute our hypothesis that no dorsal stream structure would be implicated in affective prosody recognition nor its underlying processes. The arcuate fasciculus (encapsulated within the SLF during segmentation in the current study) and SLF are typically considered dorsal language pathways [62,63], but, not surprisingly, previous research demonstrates overlap between the ventral and dorsal processing streams in the right hemisphere [37,63]. Additionally, previous work highlights emotional, semantic, and prosody recognition associations with this white matter pathway in the right hemisphere that help to corroborate current study findings. Herbert and colleagues [64] identified a potential role of the right dorsal stream in the processing of perceptual cues involved in the identification of psychological states. Regarding emotion processing, one lesion–symptom mapping study found that lesions affecting the SLF and arcuate fasciculus in patients with penetrating head injuries showed a significant effect on emotional intelligence [65]. Finally, a work by Sammler and colleagues [37] identified the SLF as a critical structure for linguistic prosody decoding, and they posited the role of the SLF as necessary for explicit labeling of the linguistic prosody, a skill that likely requires access to the semantic representation of a given prosodic contour to facilitate accurate recognition and comprehension. Thus, it is not surprising to find the dorsally situated SLF implicated in prosody recognition processes when considering the aforementioned previous studies.

Growing evidence continues to refine the function or role of different right hemisphere neural substrates within dorsal and ventral streams for affective prosodic decoding. The functional division between the dorsal and ventral stream pathways is often explained as the dorsal stream taking on a dominant role in expression and sound-to-motor mapping, supporting the processing of temporal prosody contours and auditory-motor integration [35], and the ventral stream playing a key role in processing sound-to-meaning mapping, thus facilitating stimulus perception, recognition, and comprehension [39,66,67,68]. Our findings align with these accounts indicating the right ventral stream to be heavily implicated in emotional prosody identification, but our findings also highlight the role of dorsal stream structures that support recognition processes.

**Figure 6:**
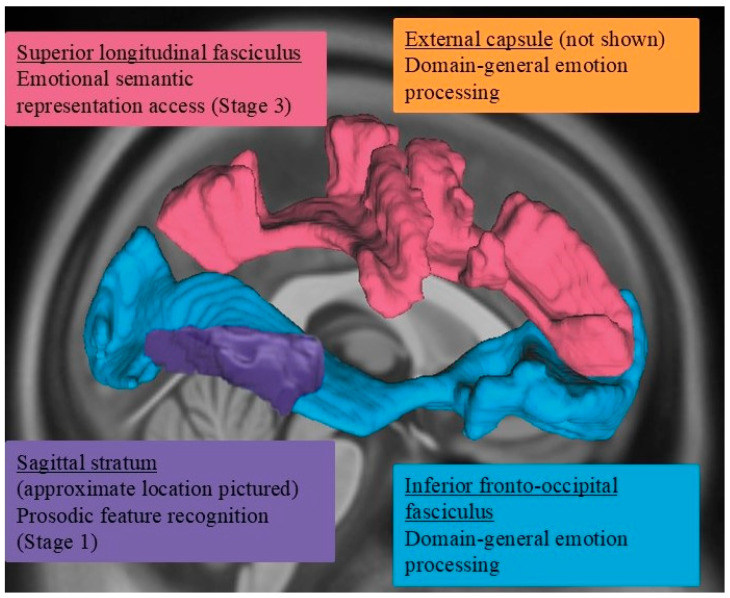
Proposed right hemisphere white matter contributions to affective prosody recognition cognitive architecture: superior longitudinal fasciculus (SLF), sagittal stratum (SS), external capsule (EC), and inferior fronto-occipital fasciculus (IFOF).

From this study, we posit that right ventral stream structures, including the IFOF and EC, to be critical for overall emotional processing abilities, both receptive and expressive, as they were implicated in affective prosody recognition and emotional facial expression recognition as well as increased perceived emotionality in speakers’ prosody [41] (see Figure 6). Furthermore, the SS appears to be involved in Stage 1 (acoustic–prosodic decoding) processing, transmitting early acoustic signals to the cortex for later integration with cognitive-linguistic prosodic information (Figure 6). Findings for Stage 2 processing, ARACCE access, were inconclusive. The UF might play a role in Stage 2 (ARACCE access) or stage 3 (emotion semantic representation access) processing based on its role in propositional language processing in the left hemisphere, particularly semantic information [69], but further research is needed to corroborate its involvement. Sihvonen and colleagues [38] observed a significant association between UF volume and affective prosody recognition *three months* post-stroke, whereas the current study investigated lesion–symptom associations at the acute stage of recovery. Both the current study and Sihvonen and colleagues [38] observed no significant association between affective prosody performance and the UF acutely, suggesting that the UF may not be uniquely playing a role in affective prosody recognition but perhaps contribute to affective prosody recognition recovery. Previous work from our group observed a significant association between UF damage and affective prosody expression measures, which we posit to indicate a role of the UF in general affective prosody processing. Finally, Stage 3 processing, involving accessing the semantic representations of an emotion, appears to be facilitated by the SLF, a dorsal stream white matter structure (Figure 6). Future work focusing on SLF integrity and its role in expressive affective prosody abilities is needed to further elucidate its part in the proposed cognitive architecture model under investigation in the current study.

Of note, the lesion–symptom associations observed in the current study were evident only when analyzing percent damage to ROIs, and no associations were observed between commonly investigated DTI measures (fractional anisotropy, mean diffusivity) of critical ROIs and behavioral performance. Given the limited number of DTI scans available per timepoint in the study sample, these results are not surprising. However, previous studies have found an increase in extracellular free water in the infarcted area at the acute stage post stroke (first 24 h), while mean diffusivity first dropped and then increased [70]. Moreover, fractional anisotropy values were also observed to drop across timepoints and were associated with Montreal Cognitive Assessment performance 30 and 90 days post stroke [70]. Thus, at acute timepoints, infarct area and increases in extracellular free water might be a better marker for the extent of white matter damage and performance impairments post stroke while fractional anisotropy and mean diffusivity show the longitudinal aftereffects of not only ischemic lesions but also microstructural white matter damage as a result of increased extracellular fluids [71].

Though this study contributed to the evidence base on white matter integrity and affective prosody recovery post-RHS, the current work is not without limitations. First, the sample size of participants, particularly those identified as aprosodic and who completed DTI scans at each timepoint, was small, limiting statistical inquiry into these brain–behavior associations. The single-variable predictor models utilized facilitated investigation of white matter tract integrity and behavioral performance of prosody tasks acutely with a small sample and without theoretical insight from prior investigations on specific covariates to support use of multivariable models. The correction for multiple comparisons employed helped to control Type I and II error rates. Our findings pertaining to white matter tract integrity and prosody subprocess are novel and will be confirmed in a follow-up study with a new and larger participant sample. With a larger pool of participants, a division of the sample into an aprosodic and non-aprosodic group could provide a more detailed depiction of the recovery and white matter integrity associations for different aprosodia subtypes throughout the first year of recovery. Such a study of specific behavioral impairment locus and longitudinal recovery is the focus of another ongoing investigation. Use of neuroimaging corpus data with affective prosody measures may allow finer analysis of DTI metrics and affective prosody recognition performance, but to the best of our knowledge, such corpus does not exist at this time.

Additionally, participant assessment focused heavily on prosody and emotion, which was the focus of the current investigation, but lacked consistent assessment of other cognitive skills, such as memory and attention, which likely influence prosody recovery post-RHS similar to other aspects of communication recovery post-stroke [72,73]. Future studies should include a mixture of assessments that focus on cognitive, communicative, and emotional skills to refine understanding of their unique and combined contributions to affective prosody recovery. Inclusion of cognitive measures would also aid in teasing apart behavioral task performance improvement due to recovery or practice. Since significant improvement on the prosody battery was not observed consistently across timepoints and tasks, interpretation of the significant improvement in affective prosody recognition from acute to chronic testing suggests a recovery rather than a repeated exposure/practice mechanism.

## 5. Conclusions

This study replicated previous findings [38] and added to pre-existing affective prosody models by investigating long-term behavioral recovery after right hemisphere stroke and highlighting the involvement of specific white matter structures in affective prosody recognition and its underlying, supportive skills. Sheppard and colleagues [8] suggested that a large portion of patients displaying acute deficits in emotional receptive prosody will experience long-term affective receptive aprosodia, underscoring the need for speech therapy services targeting these paralinguistic deficits. However, as highlighted in a review by Stockbridge and colleagues [74], there has been a scarcity of research into interventions for communication dysfunctions post-RHS outside of cognitive deficits (see [75] for a review), and, to date, only one study has directly investigated intervention for receptive affective aprosodia in this population [23]. Thus, this project accentuates the need for more research into receptive affective aprosodia post-RHS that includes larger sample sizes and participants with varying severities and symptomatology. Moreover, if patients are followed longitudinally through their stroke recovery, using tractography-based analysis to map acute changes in white matter architecture and detailing the changes in white matter structure and integrity over time, findings could provide insights into how white matter architecture degenerates or rewires post-stroke.

Discourse relies not only on linguistic but also on paralinguistic components for successful communicative exchanges between/among interlocutors. The ability to quickly and accurately decode and integrate both linguistic and prosodic information is crucial, especially when these components appear to be in conflict (i.e., sarcasm). By elucidating the neural correlates of receptive affective aprosodia and related skills, the breadth and depth of detection and intervention options can be expanded for clinicians, particularly speech-language pathologists, to support rehabilitation efforts and improve patients’ quality of life through individualized, evidence-based methods.

## Figures and Tables

**Table 1:** Participants’ percent accuracy mean ± standard deviation among tasks across timepoints.

Task	Acute (*n* = 24)	Subacute (*n* = 13)	Chronic (*n* = 23)	Controls (*n* = 57)
Word prosody recognition	64.58 *^†^ ± 14.90	71.43 * ± 12.43	71.56 ^†^ ± 12.97	77.9 ± 8.19
Recognition of prosodic features (Stage 1)	77.43 * ± 16.62	85.86 ± 11.15	82.25 ± 16.96	87.96 ± 11.24
Matching features with emotions (Stage 2)	68.84 ± 14.90	72.62 ± 15.48	75.00 ± 13.76	75.69 ± 11.64
Emotion synonym matching (Stage 3)	90.87 * ± 7.20	90.77 * ± 8.98	92.61 * ± 5.74	96.76 ± 4.47
Emotional facial expression recognition (domain-general)	83.42 * ± 10.04	88.39 ± 9.59	85.34 ± 12.13	89.79 ± 5.71

* Indicates scores from RHS participants significantly differed from scores of healthy control participants in linear mixed effects models reported in Section 3.1 (*p* < 0.05). ^†^ Indicates scores that statistically differed between timepoints for RHS participants in linear mixed effects models reported in Section 3.2 (*p* < 0.05).

**Table 2:** Task cut-off scores and percentage of RHS participants classified as impaired by task across timepoints.

Task	Cut-Off Score (%)	Impaired: Acute (%)	Impaired: Subacute (%)	Impaired: Chronic (%)
Word prosody recognition	63.33	41.67	14.29	21.74
Recognition of prosodic features (Stage 1)	64.58	33.33	7.14	21.74
Matching features with emotion (Stage 2)	58.33	29.17	21.43	21.74
Emotion synonym matching (Stage 3)	87.50	33.33	21.43	21.74
Emotional facial expression recognition (domain-general emotion)	80.00	29.17	28.57	34.78

*Note*. Impairment cut-off scores for each task is based on the 5th percentile of scores from healthy matched controls.

**Table 3:** Significant models for word prosody recognition accuracy.

Model	IV	Estimate	SE	*t*	*p*
Base	(Intercept)	45.568	12.683	3.593	0.002
	age	−0.460	0.192	−2.390	0.027
	education	2.802	0.742	3.778	0.001
Base + WML:EC	(Intercept)	38.582	16.069	2.401	0.027
	acute lesion volume	0.117	0.121	0.965	0.347
	EC	−0.316	0.136	−2.325	0.032
	age	−0.452	0.270	−1.676	0.111
	education	3.297	0.845	3.900	0.001
Base + WML:IFOF	(Intercept)	41.351	15.857	2.608	0.018
	acute lesion volume	0.039	0.075	0.526	0.606
	IFOF	−0.237	0.098	−2.408	0.027
	age	−0.495	0.156	−3.168	0.005
	education	3.254	0.980	3.322	0.004
Base + WML:SLF	(Intercept)	43.233	14.569	2.967	0.008
	acute lesion volume	−0.097	0.079	−1.229	0.235
	SLF	0.169	0.069	2.443	0.025
	age	−0.428	0.271	−1.575	0.133
	education	2.872	0.826	3.476	0.003

Note. IV = Independent variable; SE = Standard error; WML = White matter lesion; EC = External capsule; IFOF = Inferior fronto-occipital fasciculus; SLF = Superior longitudinal fasciculus.

**Table 4:** Significant models for recognition of prosodic features (Stage 1) accuracy.

Model	IV	Estimate	SE	*t*	*p*
Base	(Intercept)	70.268	20.266	3.467	0.002
	age	−0.217	0.236	−0.918	0.370
	education	1.218	1.078	1.130	0.272
Base + WML:SLF	(Intercept)	81.446	6.214	13.107	<0.001
	acute lesion volume	−0.163	0.169	−0.962	0.347
	SLF	−2.068	0.973	−2.127	0.046
Base + WML:UF	(Intercept)	77.607	4.547	17.067	<0.001
	acute lesion volume	−0.130	0.075	−1.731	0.098
	UF	0.294	0.085	3.456	0.002
Base + WML:SS	(Intercept)	83.352	6.184	13.478	<0.001
	acute lesion volume	−0.101	0.121	−0.842	0.409
	SS	−3.373	0.782	−4.312	<0.001
Base + WML:BCC	(Intercept)	76.241	4.816	15.832	<0.001
	acute lesion volume	0.047	0.089	0.532	0.600
	BCC	0.462	0.151	3.062	0.006
Base + WML:GCC	(Intercept)	76.226	4.813	15.836	<0.001
	acute lesion volume	0.048	0.088	0.546	0.591
	GCC	24.178	7.582	3.189	0.004

Note. IV = Independent variable; SE = Standard error; WML = White matter lesion; SLF = Superior longitudinal fasciculus; UF = Uncinate fasciculus; SS = Sagittal stratum; BCC = Body of the corpus callosum; GCC = Genu of the corpus callosum.

**Table 5:** Significant models for matching features with emotions (Stage 2) accuracy.

Model	IV	Estimate	SE	*t*	*p*
Base	(Intercept)	48.369	15.421	3.137	0.005
	age	−0.315	0.118	−2.682	0.015
	education	2.446	0.911	2.685	0.015
Base + WML:SLF	(Intercept)	41.152	13.440	3.062	0.007
	acute lesion volume	−0.102	0.072	−1.403	0.179
	SLF	0.328	0.078	4.211	0.001
	age	−0.158	0.098	−1.611	0.126
	education	2.318	0.986	2.350	0.031

Note. IV = Independent variable; SE = Standard error; WML = White matter lesion; SLF = Superior longitudinal fasciculus.

**Table 6:** Significant models for emotion synonym matching (Stage 3) accuracy.

Model	IV	Estimate	SE	*t*	*p*
Base	(Intercept)	67.867	5.908	11.487	<0.001
	age	−0.001	0.062	−0.020	0.984
	education	1.469	0.314	4.685	<0.001
Base + WML:SLF	(Intercept)	67.965	5.522	12.309	<0.001
	acute lesion volume	0.027	0.021	1.316	0.207
	SLF	−0.080	0.024	−3.319	0.004
	education	1.468	0.314	4.679	<0.001
Base + WML:BCC	(Intercept)	66.286	5.473	12.112	<0.001
	acute lesion volume	−0.013	0.017	−0.774	0.450
	BCC	0.250	0.054	4.594	<0.001
	education	1.558	0.316	4.933	<0.001
Base + WML:GCC	(Intercept)	66.274	5.471	12.114	<0.001
	acute lesion volume	−0.013	0.017	−0.757	0.460
	GCC	12.995	2.746	4.732	<0.001
	education	1.558	0.316	4.935	<0.001

Note. IV = Independent variable; SE = Standard error; WML = White matter lesion; SLF = Superior longitudinal fasciculus; BCC = Body of the corpus callosum; GCC = Genu of the corpus callosum.

**Table 7:** Significant models for emotional facial expression recognition (domain-general emotion recognition) accuracy.

Model	IV	Estimate	SE	*t*	*p*
Base	(Intercept)	65.169	7.786	8.370	<0.001
	age	−0.062	0.133	−0.464	0.649
	education	1.347	0.543	2.479	0.026
Base + WML:EC	(Intercept)	59.538	8.281	7.190	<0.001
	acute lesion volume	0.184	0.058	3.178	0.007
	EC	−0.261	0.101	−2.594	0.021
	education	1.414	0.494	2.859	0.013
Base + WML:IFOF	(Intercept)	60.159	7.867	7.647	<0.001
	acute lesion volume	0.128	0.035	3.647	0.003
	IFOF	−0.213	0.068	−3.132	0.007
	education	1.389	0.488	2.845	0.013

Note. IV = Independent variable; SE = Standard error; WML = White matter lesion; EC = External capsule; IFOF = Inferior fronto-occipital fasciculus.

## Data Availability

Analysis files and deidentified data are stored in a data repository at https://archive.data.jhu.edu/. Behavioral testing materials can be found at https://score.jhmi.edu/downloads.html (accessed on 20 December 2024).

## Supplementary Materials

The following supporting information can be downloaded at: https://www.mdpi.com/article/10.3390/brainsci15070769/s1, Table S1. Nonsignificant models for acute Word Prosody Recognition (affective prosody recognition); Table S2. Nonsignificant models for acute Recognition of Prosodic Features (Stage 1); Table S3. Nonsignificant models for acute Matching Features to Emotions (Stage 2); Table S4. Nonsignificant models for acute Emotion Synonym Matching (Stage 3); Table S5. Nonsignificant models for acute Emotional Facial Expression Recognition (domain-general emotion recognition); Table S6. Analysis of variance comparing full covariate + lesion models to base models for acute Word Prosody Recognition (affective prosody recognition) in RHS participants; Table S7. Analysis of variance comparing full covariate + lesion models to base models for acute Recognition of Prosodic Features (Stage 1) task in RHS participants; Table S8. Analysis of variance comparing full covariate + lesion models to base models for acute Matching Features to Emotion (Stage 2) task in RHS participants; Table S9. Analysis of variance comparing full covariate + lesion models to base models for acute Emotion Synonym Matching (Stage 3) task in RHS participants; Table S10. Analysis of variance comparing full covariate + lesion models to base models for acute Emotional Facial Expression Recognition (domain-general emotion) task in RHS participants.

## Abbreviations

The following abbreviations are used in this manuscript:

RHS	Right hemisphere stroke
ARACCE	Abstract representations of acoustic characteristics that convey emotion
MRI	Magnetic resonance imaging
DTI	Diffusion tensor imaging
DWI	Diffusion weighted imaging
MNI	Montreal Neurological Institute
SPM	Statistical parametric mapping
JHU	Johns Hopkins University
ROI	Regions of interest
FDR	False discovery rate
EC	External capsule
IFOF	Inferior fronto-occipital fasciculus
UF	Uncinate fasciculus
SLF	Superior longitudinal fasciculus
SS	Sagittal stratum
BCC	Body of the corpus callosum
GCC	Genu of the corpus callosum

## References

[1] Wymer J.H., Lindman Linda S., Booksh R.L. (2002). A Neuropsychological Perspective of Aprosody: Features, Function, Assessment, and Treatment. Appl. Neuropsychol..

[2] Schirmer A., Kotz S.A. (2006). Beyond the Right Hemisphere: Brain Mechanisms Mediating Vocal Emotional Processing. Trends Cogn. Sci..

[3] Blonder L.X., Pettigrew L.C., Kryscio R.J. (2012). Emotion Recognition and Marital Satisfaction in Stroke. J. Clin. Exp. Neuropsychol..

[4] Hillis A.E., Tippett D.C. (2014). Stroke Recovery: Surprising Influences and Residual Consequences. Adv. Med..

[5] Martinez M., Multani N., Anor C.J., Misquitta K., Tang-Wai D.F., Keren R., Fox S., Lang A.E., Marras C., Tartaglia M.C. (2018). Emotion Detection Deficits and Decreased Empathy in Patients with Alzheimer’s Disease and Parkinson’s Disease Affect Caregiver Mood and Burden. Front. Aging Neurosci..

[6] O’Connell K., Marsh A.A., Edwards D.F., Dromerick A.W., Seydell-Greenwald A. (2022). Emotion Recognition Impairments and Social Well-Being Following Right-Hemisphere Stroke. Neuropsychol. Rehabil..

[7] Dara C., Bang J., Gottesman R.F., Hillis A.E. (2014). Right Hemisphere Dysfunction Is Better Predicted by Emotional Prosody Impairments as Compared to Neglect. J. Neurol. Transl. Neurosci..

[8] Sheppard S.M., Keator L.M., Breining B.L., Wright A.E., Saxena S., Tippett D.C., Hillis A.E. (2020). Right Hemisphere Ventral Stream for Emotional Prosody Identification: Evidence from Acute Stroke. Neurology.

[9] Sheppard S.M., Stockbridge M.D., Keator L.M., Murray L.L., Blake M.L. (2022). Right Hemisphere Damage working group, Evidence-Based Clinical Research Committee, Academy of Neurologic Communication Disorders and Sciences The Company Prosodic Deficits Keep Following Right Hemisphere Stroke: A Systematic Review. J. Int. Neuropsychol. Soc..

[10] Ukaegbe O.C., Holt B.E., Keator L.M., Brownell H., Blake M.L., Lundgren K. (2022). Aprosodia Following Focal Brain Damage: What’s Right and What’s Left?. Am. J. Speech-Lang. Pathol..

[11] Coulombe V., Joyal M., Martel-Sauvageau V., Monetta L. (2023). Affective Prosody Disorders in Adults with Neurological Conditions: A Scoping Review. Int. J. Lang. Commun. Disord..

[12] Hewetson R., Cornwell P., Shum D.H.K. (2021). Relationship and Social Network Change in People With Impaired Social Cognition Post Right Hemisphere Stroke. Am. J. Speech Lang. Pathol..

[13] Blake M.L., Duffy J.R., Myers P.S., Tompkins C.A. (2002). Prevalence and Patterns of Right Hemisphere Cognitive/Communicative Deficits: Retrospective Data from an Inpatient Rehabilitation Unit. Aphasiology.

[14] Leigh R., Oishi K., Hsu J., Lindquist M., Gottesman R.F., Jarso S., Crainiceanu C., Mori S., Hillis A.E. (2013). Acute Lesions That Impair Affective Empathy. Brain.

[15] Ramsey A., Blake M.L. (2020). Speech-Language Pathology Practices for Adults With Right Hemisphere Stroke: What Are We Missing?. Am. J. Speech Lang. Pathol..

[16] Ethofer T., Anders S., Erb M., Herbert C., Wiethoff S., Kissler J., Grodd W., Wildgruber D. (2006). Cerebral Pathways in Processing of Affective Prosody: A Dynamic Causal Modeling Study. Neuroimage.

[17] Seydell-Greenwald A., Chambers C.E., Ferrara K., Newport E.L. (2020). What You Say versus How You Say It: Comparing Sentence Comprehension and Emotional Prosody Processing Using fMRI. NeuroImage.

[18] Sheppard S.M., Meier E.L., Zezinka Durfee A., Walker A., Shea J., Hillis A.E. (2021). Characterizing Subtypes and Neural Correlates of Receptive Aprosodia in Acute Right Hemisphere Stroke. Cortex.

[19] Wright A., Saxena S., Sheppard S.M., Hillis A.E. (2018). Selective Impairments in Components of Affective Prosody in Neurologically Impaired Individuals. Brain Cogn..

[20] Bowers D., Bauer R.M., Heilman K.M. (1993). The Nonverbal Affect Lexicon: Theoretical Perspectives from Neuropsychological Studies of Affect Perception. Neuropsychology.

[21] Ross E.D. (1981). The Aprosodias. Functional-Anatomic Organization of the Affective Components of Language in the Right Hemisphere. Arch. Neurol..

[22] Gorelick P.B., Ross E.D. (1987). The Aprosodias: Further Functional-Anatomical Evidence for the Organisation of Affective Language in the Right Hemisphere. J. Neurol. Neurosurg. Psychiatry.

[23] Durfee A.Z., Sheppard S.M., Meier E.L., Bunker L., Cui E., Crainiceanu C., Hillis A.E. (2021). Explicit Training to Improve Affective Prosody Recognition in Adults with Acute Right Hemisphere Stroke. Brain Sci..

[24] Belyk M., Brown S. (2014). Perception of Affective and Linguistic Prosody: An ALE Meta-Analysis of Neuroimaging Studies. Soc. Cogn. Affect. Neurosci..

[25] Themistocleous C. (2025). Linguistic and Emotional Prosody: A Systematic Review and ALE Meta-Analysis. Neurosci. Biobehav. Rev..

[26] Mitchell R.L.C., Elliott R., Barry M., Cruttenden A., Woodruff P.W.R. (2003). The Neural Response to Emotional Prosody, as Revealed by Functional Magnetic Resonance Imaging. Neuropsychologia.

[27] Wildgruber D., Riecker A., Hertrich I., Erb M., Grodd W., Ethofer T., Ackermann H. (2005). Identification of Emotional Intonation Evaluated by fMRI. Neuroimage.

[28] Grandjean D. (2021). Brain Networks of Emotional Prosody Processing. Emot. Rev..

[29] Ross E.D., Monnot M. (2008). Neurology of Affective Prosody and Its Functional-Anatomic Organization in Right Hemisphere. Brain Lang..

[30] Starkstein S.E., Federoff J.P., Price T.R., Leiguarda R.C., Robinson R.G. (1994). Neuropsychological and Neuroradiologic Correlates of Emotional Prosody Comprehension. Neurology.

[31] Walker J.P., Daigle T., Buzzard M. (2002). Hemispheric Specialisation in Processing Prosodic Structures: Revisited. Aphasiology.

[32] Obleser J., Eisner F., Kotz S.A. (2008). Bilateral Speech Comprehension Reflects Differential Sensitivity to Spectral and Temporal Features. J. Neurosci..

[33] Kotz S.A., Meyer M., Alter K., Besson M., von Cramon D.Y., Friederici A.D. (2003). On the Lateralization of Emotional Prosody: An Event-Related Functional MR Investigation. Brain Lang..

[34] Buchanan T.W., Lutz K., Mirzazade S., Specht K., Shah N.J., Zilles K., Jäncke L. (2000). Recognition of Emotional Prosody and Verbal Components of Spoken Language: An fMRI Study. Brain Res. Cogn. Brain Res..

[35] Hickok G., Poeppel D. (2007). The Cortical Organization of Speech Processing. Nat. Rev. Neurosci..

[36] Durfee A.Z., Sheppard S.M., Blake M.L., Hillis A.E. (2021). Lesion Loci of Impaired Affective Prosody: A Systematic Review of Evidence from Stroke. Brain Cogn..

[37] Sammler D., Grosbras M.-H., Anwander A., Bestelmeyer P.E.G., Belin P. (2015). Dorsal and Ventral Pathways for Prosody. Curr. Biol..

[38] Sihvonen A.J., Sammler D., Ripollés P., Leo V., Rodríguez-Fornells A., Soinila S., Särkämö T. (2022). Right Ventral Stream Damage Underlies Both Poststroke Aprosodia and Amusia. Eur. J. Neurol..

[39] Frühholz S., Gschwind M., Grandjean D. (2015). Bilateral Dorsal and Ventral Fiber Pathways for the Processing of Affective Prosody Identified by Probabilistic Fiber Tracking. NeuroImage.

[40] Hickok G., Poeppel D. (2004). Dorsal and Ventral Streams: A Framework for Understanding Aspects of the Functional Neuroanatomy of Language. Cognition.

[41] Patel S., Oishi K., Wright A., Sutherland-Foggio H., Saxena S., Sheppard S.M., Hillis A.E. (2018). Right Hemisphere Regions Critical for Expression of Emotion Through Prosody. Front. Neurol..

[42] Paulmann S., Ott D.V.M., Kotz S.A. (2011). Emotional Speech Perception Unfolding in Time: The Role of the Basal Ganglia. PLoS ONE.

[43] Kaplan E., Goodglass H., Weintraub S. (2001). The Boston Naming Test.

[44] Berube S., Nonnemacher J., Demsky C., Glenn S., Saxena S., Wright A., Tippett D.C., Hillis A.E. (2019). Stealing Cookies in the Twenty-First Century: Measures of Spoken Narrative in Healthy Versus Speakers With Aphasia. Am. J. Speech-Lang. Pathol..

[45] Goodglass H., Kaplan E., Barresi B. (2001). Boston Diagnostic Aphasia Examination.

[46] Ota H., Fujii T., Suzuki K., Fukatsu R., Yamadori A. (2001). Dissociation of Body-Centered and Stimulus-Centered Representations in Unilateral Neglect. Neurology.

[47] Banse R., Scherer K.R. (1996). Acoustic Profiles in Vocal Emotion Expression. J. Pers. Soc. Psychol..

[48] Rorden C., Bonilha L., Fridriksson J., Bender B., Karnath H.-O. (2012). Age-Specific CT and MRI Templates for Spatial Normalization. Neuroimage.

[49] Oishi K., Faria A., Jiang H., Li X., Akhter K., Zhang J., Hsu J.T., Miller M.I., van Zijl P.C.M., Albert M. (2009). Atlas-Based Whole Brain White Matter Analysis Using Large Deformation Diffeomorphic Metric Mapping: Application to Normal Elderly and Alzheimer’s Disease Participants. NeuroImage.

[50] Jiang H., van Zijl P.C.M., Kim J., Pearlson G.D., Mori S. (2006). DtiStudio: Resource Program for Diffusion Tensor Computation and Fiber Bundle Tracking. Comput. Methods Programs Biomed..

[51] Pumphrey J.D., Ramani S., Islam T., Berard J.A., Seegobin M., Lymer J.M., Freedman M.S., Wang J., Walker L.A.S. (2024). Assessing Multimodal Emotion Recognition in Multiple Sclerosis with a Clinically Accessible Measure. Mult. Scler. Relat. Disord..

[52] Kraemer M., Herold M., Uekermann J., Kis B., Daum I., Wiltfang J., Berlit P., Diehl R.R., Abdel-Hamid M. (2013). Perception of Affective Prosody in Patients at an Early Stage of Relapsing-Remitting Multiple Sclerosis. J. Neuropsychol..

[53] Thompson W.F., Marin M.M., Stewart L. (2012). Reduced Sensitivity to Emotional Prosody in Congenital Amusia Rekindles the Musical Protolanguage Hypothesis. Proc. Natl. Acad. Sci. USA.

[54] Catani M., Thiebaut de Schotten M. (2008). A Diffusion Tensor Imaging Tractography Atlas for Virtual in Vivo Dissections. Cortex.

[55] Wu Y., Sun D., Wang Y., Wang Y. (2016). Subcomponents and Connectivity of the Inferior Fronto-Occipital Fasciculus Revealed by Diffusion Spectrum Imaging Fiber Tracking. Front. Neuroanat..

[56] Ethofer T., Bretscher J., Wiethoff S., Bisch J., Schlipf S., Wildgruber D., Kreifelts B. (2013). Functional Responses and Structural Connections of Cortical Areas for Processing Faces and Voices in the Superior Temporal Sulcus. NeuroImage.

[57] Pierce J.E., Péron J. (2020). The Basal Ganglia and the Cerebellum in Human Emotion. Soc. Cogn. Affect. Neurosci..

[58] Efthymiopoulou E., Kasselimis D.S., Ghika A., Kyrozis A., Peppas C., Evdokimidis I., Petrides M., Potagas C. (2017). The Effect of Cortical and Subcortical Lesions on Spontaneous Expression of Memory-Encoded and Emotionally Infused Information: Evidence for a Role of the Ventral Stream. Neuropsychologia.

[59] Maldonado I.L., Destrieux C., Ribas E.C., Siqueira de Abreu Brito Guimarães B., Cruz P.P., Duffau H. (2021). Composition and Organization of the Sagittal Stratum in the Human Brain: A Fiber Dissection Study. J. Neurosurg..

[60] Davis C., Oishi K., Faria A., Hsu J., Gomez Y., Mori S., Hillis A.E. (2016). White Matter Tracts Critical for Recognition of Sarcasm. Neurocase.

[61] Shahid H., Sebastian R., Schnur T.T., Hanayik T., Wright A., Tippett D.C., Fridriksson J., Rorden C., Hillis A.E. (2017). Important Considerations in Lesion-Symptom Mapping: Illustrations from Studies of Word Comprehension. Hum. Brain Mapp..

[62] Rauschecker J.P., Scott S.K. (2009). Maps and Streams in the Auditory Cortex: Nonhuman Primates Illuminate Human Speech Processing. Nat. Neurosci..

[63] Zündorf I.C., Lewald J., Karnath H.-O. (2016). Testing the Dual-Pathway Model for Auditory Processing in Human Cortex. Neuroimage.

[64] Herbet G., Lafargue G., Bonnetblanc F., Moritz-Gasser S., Menjot de Champfleur N., Duffau H. (2014). Inferring a Dual-Stream Model of Mentalizing from Associative White Matter Fibres Disconnection. Brain.

[65] Barbey A.K., Colom R., Grafman J. (2014). Distributed Neural System for Emotional Intelligence Revealed by Lesion Mapping. Soc. Cogn. Affect. Neurosci..

[66] Fridriksson J., Yourganov G., Bonilha L., Basilakos A., Den Ouden D.-B., Rorden C. (2016). Revealing the Dual Streams of Speech Processing. Proc. Natl. Acad. Sci. USA.

[67] Friederici A.D. (2011). The Brain Basis of Language Processing: From Structure to Function. Physiol. Rev..

[68] Saur D., Kreher B.W., Schnell S., Kümmerer D., Kellmeyer P., Vry M.-S., Umarova R., Musso M., Glauche V., Abel S. (2008). Ventral and Dorsal Pathways for Language. Proc. Natl. Acad. Sci. USA.

[69] Zhang J., Zhong S., Zhou L., Yu Y., Tan X., Wu M., Sun P., Zhang W., Li J., Cheng R. (2021). Correlations between Dual-Pathway White Matter Alterations and Language Impairment in Patients with Aphasia: A Systematic Review and Meta-Analysis. Neuropsychol. Rev..

[70] Kern K.C., Wright C.B., Leigh R. (2022). Global Changes in Diffusion Tensor Imaging during Acute Ischemic Stroke and Post-Stroke Cognitive Performance. J. Cereb. Blood Flow. Metab..

[71] Yu X., Yin X., Hong H., Wang S., Jiaerken Y., Zhang F., Pasternak O., Zhang R., Yang L., Lou M. (2021). Increased Extracellular Fluid Is Associated with White Matter Fiber Degeneration in CADASIL: In Vivo Evidence from Diffusion Magnetic Resonance Imaging. Fluids Barriers CNS.

[72] Harnish S.M., Schwen Blackett D., Zezinka A., Lundine J.P., Pan X. (2018). Influence of Working Memory on Stimulus Generalization in Anomia Treatment: A Pilot Study. J. Neurolinguist..

[73] Harnish S.M., Lundine J.P. (2015). Nonverbal Working Memory as a Predictor of Anomia Treatment Success. Am. J. Speech-Lang. Pathol..

[74] Stockbridge M.D., Sheppard S.-M., Keator L.M., Murray L.L., Blake M.L., Right Hemisphere Disorders Working Group, Evidence-Based Clinical Research Committee, Academy of Neurological Communication Disorders and Sciences (2021). Aprosodia Subsequent to Right Hemisphere Brain Damage: A Systematic Review and Meta-Analysis. J. Int. Neuropsychol. Soc..

[75] Lehman Blake M., Frymark T., Venedictov R. (2013). An Evidence-Based Systematic Review on Communication Treatments for Individuals with Right Hemisphere Brain Damage. Am. J. Speech Lang. Pathol..

