# Supplementary material for: Elucidating White Matter Contributions to the Cognitive Architecture of Affective Prosody Recognition: Evidence from Right Hemisphere Stroke
Source: Brain Sci. 2025 Jul 19;15(7):769. doi: 10.3390/brainsci15070769 (PMC12293220; doi:10.3390/brainsci15070769)
Supplement: Supplementary file 1 [file brainsci-15-00769-s001.zip › brainsci-3689451-supplementary.pdf]

Supplementary Materials

Table S1. Nonsignificant models for acute Word Prosody Recognition (affective prosody recognition).

Model	Predictor	Estimate	*SE*	*t*-Value	*p*-Value
Intercept-only	(Intercept)	65.475	3.464	18.900	<0.001
Base	(Intercept)	45.568	12.683	3.593	0.002
	age	-0.460	0.192	-2.390	0.027
	education	2.802	0.742	3.778	0.001
Base + WML:EC	(Intercept)	38.582	16.069	2.401	0.027
	lesion volume	0.117	0.121	0.965	0.347
	EC	-0.316	0.136	-2.325	0.032
	age	-0.452	0.270	-1.676	0.111
	education	3.297	0.845	3.900	0.001
Base + WML:IFOF	(Intercept)	41.351	15.857	2.608	0.018
	lesion volume	0.039	0.075	0.526	0.606
	IFOF	-0.237	0.098	-2.408	0.027
	age	-0.495	0.156	-3.168	0.005
	education	3.254	0.980	3.322	0.004
Base + WML:SLF	(Intercept)	43.233	14.569	2.967	0.008
	lesion volume	-0.097	0.079	-1.229	0.235
	SLF	0.169	0.069	2.443	0.025
	age	-0.428	0.271	-1.575	0.133
	education	2.872	0.826	3.476	0.003
Base + WML:UF	(Intercept)	38.517	16.184	2.380	0.029
	lesion volume	0.128	0.143	0.898	0.381
	UF	-0.230	0.136	-1.693	0.108
	age	-0.415	0.259	-1.601	0.127
	education	3.097	0.800	3.870	0.001
Base + WML:BCC	(Intercept)	43.643	15.336	2.846	0.011
	lesion volume	-0.012	0.078	-0.159	0.875
	BCC	0.207	0.155	1.339	0.197
	age	-0.445	0.272	-1.637	0.119
	education	2.869	0.818	3.506	0.003
Base + WML:SS	(Intercept)	46.034	14.507	3.173	0.005
	lesion volume	0.000	0.124	0.003	0.998
	SS	-0.179	0.929	-0.193	0.849
	age	-0.468	0.249	-1.879	0.077
	education	2.827	0.790	3.579	0.002
Base + WML:GCC	(Intercept)	43.806	15.323	2.859	0.010
	lesion volume	-0.013	0.078	-0.162	0.873
	GCC	10.204	7.819	1.305	0.208
	age	-0.447	0.271	-1.649	0.116
	education	2.868	0.817	3.509	0.003

Note. Lesion volume calculated at acute stage. SE = Standard Error; WML = white matter lesion; EC = external capsule; IFOF = inferior fronto-occipital fasciculus; SLF = superior longitudinal fasciculus; UF = uncinate fasciculus; BCC = body of the corpus callosum; SS = sagittal stratum; GCC = genu of the corpus callosum.

Table S2. Nonsignificant models for acute Recognition of Prosodic Features (Stage 1).

Model	Predictor	Estimate	*SE*	*t*-Value	*p*-Value
Intercept-only	(Intercept)	78.047	4.341	17.980	<0.001
Base	(Intercept)	70.268	20.266	3.467	0.002
	age	-0.217	0.236	-0.918	0.370
	education	1.218	1.078	1.130	0.272
Base + WML:EC	(Intercept)	76.822	4.701	16.342	<0.001
	lesion volume	0.010	0.141	0.070	0.945
	EC	0.082	0.263	0.314	0.757
Base + WML:IFOF	(Intercept)	77.117	4.799	16.070	<0.001
	lesion volume	0.071	0.103	0.690	0.498
	IFOF	-0.086	0.158	-0.546	0.591
Base + WML:SLF	(Intercept)	81.446	6.214	13.107	<0.001
	lesion volume	-0.163	0.169	-0.962	0.347
	SLF	-2.068	0.973	-2.127	0.046
Base + WML:UF	(Intercept)	77.607	4.547	17.067	<0.001
	lesion volume	-0.130	0.075	-1.731	0.098
	UF	0.294	0.085	3.456	0.002
Base + WML:BCC	(Intercept)	76.241	4.816	15.832	<0.001
	lesion volume	0.047	0.089	0.532	0.600
	BCC	0.462	0.151	3.062	0.006
Base + WML:SS	(Intercept)	83.352	6.184	13.478	<0.001
	lesion volume	-0.101	0.121	-0.842	0.409
	SS	-3.373	0.782	-4.312	0.000
Base + WML:GCC	(Intercept)	76.226	4.813	15.836	<0.001
	lesion volume	0.048	0.088	0.546	0.591
	GCC	24.178	7.582	3.189	0.004

Note. Lesion volume calculated at acute stage. SE = Standard Error; WML = white matter lesion; EC = external capsule; IFOF = inferior fronto-occipital fasciculus; SLF = superior longitudinal fasciculus; UF = uncinate fasciculus; BCC = body of the corpus callosum; SS = sagittal stratum; GCC = genu of the corpus callosum.

Table S3. Nonsignificant models for acute Matching Features to Emotions (Stage 2).

Model	Predictor	Estimate	*SE*	*t*-Value	*p*-Value
Intercept-only	(Intercept)	69.274	3.176	21.810	<0.001
Base	(Intercept)	48.369	15.421	3.137	0.005
	age	-0.315	0.118	-2.682	0.015
	education	2.446	0.911	2.685	0.015
Base + WML:EC	(Intercept)	41.692	16.992	2.454	0.025
	lesion volume	0.124	0.117	1.064	0.302
	EC	-0.201	0.210	-0.959	0.351
	age	-0.267	0.119	-2.248	0.038
	education	2.660	1.136	2.342	0.032
Base + WML:IFOF	(Intercept)	43.414	15.915	2.728	0.014
	lesion volume	0.080	0.065	1.232	0.235
	IFOF	-0.166	0.120	-1.382	0.185
	age	-0.285	0.124	-2.308	0.034
	education	2.621	1.112	2.356	0.031
Base + WML:SLF	(Intercept)	41.152	13.440	3.062	0.007
	lesion volume	-0.102	0.072	-1.403	0.179
	SLF	0.328	0.078	4.211	0.001
	age	-0.158	0.098	-1.611	0.126
	education	2.318	0.986	2.350	0.031
Base + WML:UF	(Intercept)	48.603	16.125	3.014	0.008
	lesion volume	-0.002	0.123	-0.016	0.988
	UF	0.066	0.151	0.435	0.669
	age	-0.280	0.129	-2.181	0.043
	education	2.259	0.983	2.297	0.035
Base + WML:BCC	(Intercept)	47.174	16.533	2.853	0.011
	lesion volume	0.037	0.067	0.547	0.591
	BCC	-0.039	0.171	-0.230	0.821
	age	-0.279	0.145	-1.925	0.071
	education	2.343	0.932	2.514	0.022
Base + WML:SS	(Intercept)	45.125	14.559	3.099	0.007
	lesion volume	0.145	0.141	1.030	0.318
	SS	-0.793	0.798	-0.994	0.334
	age	-0.193	0.137	-1.408	0.177
	education	2.122	0.914	2.321	0.033
Base + WML:GCC	(Intercept)	47.302	16.520	2.863	0.011
	lesion volume	0.036	0.067	0.540	0.596
	GCC	-2.542	8.654	-0.294	0.773
	age	-0.281	0.145	-1.937	0.070
	education	2.342	0.933	2.511	0.023

Note. Lesion volume calculated at acute stage. SE = Standard Error; WML = white matter lesion; EC = external capsule; IFOF = inferior fronto-occipital fasciculus; SLF = superior longitudinal fasciculus; UF = uncinate fasciculus; BCC = body of the corpus callosum; SS = sagittal stratum; GCC = genu of the corpus callosum.

Table S4. Nonsignificant models for acute Emotion Synonym Matching (Stage 3).

Model	Predictor	Estimate	*SE*	*t*-Value	*p*-Value
Intercept-only	(Intercept)	91.254	1.698	53.700	<0.001
Base	(Intercept)	67.867	5.908	11.487	<0.001
	age	-0.001	0.062	-0.020	0.984
	education	1.469	0.314	4.685	<0.001
Base + WML:EC	(Intercept)	67.353	8.161	8.253	<0.001
	lesion volume	-0.005	0.083	-0.056	0.956
	EC	-0.023	0.173	-0.135	0.895
	education	1.523	0.460	3.313	0.004
Base + WML:IFOF	(Intercept)	67.469	6.441	10.475	<0.001
	lesion volume	-0.011	0.028	-0.380	0.709
	IFOF	-0.013	0.069	-0.192	0.850
	education	1.510	0.369	4.094	0.001
Base + WML:SLF	(Intercept)	67.965	5.522	12.309	<0.001
	lesion volume	0.027	0.021	1.316	0.207
	SLF	-0.080	0.024	-3.319	0.004
	education	1.468	0.314	4.679	<0.001
Base + WML:UF	(Intercept)	69.131	6.763	10.222	<0.001
	lesion volume	-0.040	0.033	-1.216	0.242
	UF	0.043	0.040	1.083	0.295
	education	1.420	0.364	3.896	0.001
Base + WML:BCC	(Intercept)	66.286	5.473	12.112	<0.001
	lesion volume	-0.013	0.017	-0.774	0.450
	BCC	0.250	0.054	4.594	<0.001
	education	1.558	0.316	4.933	<0.001
Base + WML:SS	(Intercept)	68.429	5.509	12.421	<0.001
	lesion volume	0.018	0.056	0.322	0.752
	SS	-0.295	0.417	-0.707	0.490
	education	1.438	0.322	4.470	<0.001
Base + WML:GCC	(Intercept)	66.274	5.471	12.114	<0.001
	lesion volume	-0.013	0.017	-0.757	0.460
	GCC	12.995	2.746	4.732	<0.001
	education	1.558	0.316	4.935	<0.001

Note. Lesion volume calculated at acute stage. SE = Standard Error; WML = white matter lesion; EC = external capsule; IFOF = inferior fronto-occipital fasciculus; SLF = superior longitudinal fasciculus; UF = uncinate fasciculus; BCC = body of the corpus callosum; SS = sagittal stratum; GCC = genu of the corpus callosum.

Table S5. Nonsignificant models for acute Emotional Facial Expression Recognition (domain-general emotion recognition).

Model	Predictor	Estimate	*SE*	*t*-Value	*p*-Value
Intercept-only	(Intercept)	83.590	2.390	34.980	<0.001
Base	(Intercept)	65.169	7.786	8.370	<0.001
	age	-0.062	0.133	-0.464	0.649
	education	1.347	0.543	2.479	0.026
Base + WML:EC	(Intercept)	59.538	8.281	7.190	<0.001
	lesion volume	0.184	0.058	3.178	0.007
	EC	-0.261	0.101	-2.594	0.021
	education	1.414	0.494	2.859	0.013
Base + WML:IFOF	(Intercept)	60.159	7.867	7.647	<0.001
	lesion volume	0.128	0.035	3.647	0.003
	IFOF	-0.213	0.068	-3.132	0.007
	education	1.389	0.488	2.845	0.013
Base + WML:SLF	(Intercept)	63.475	8.694	7.301	<0.001
	lesion volume	0.045	0.060	0.754	0.463
	SLF	0.052	0.067	0.776	0.451
	education	1.151	0.582	1.978	0.068
Base + WML:UF	(Intercept)	61.169	8.398	7.284	<0.001
	lesion volume	0.172	0.074	2.304	0.037
	UF	-0.169	0.101	-1.677	0.116
	education	1.286	0.517	2.487	0.026
Base + WML:BCC	(Intercept)	63.965	9.063	7.057	<0.001
	lesion volume	0.073	0.039	1.897	0.079
	BCC	-0.011	0.104	-0.106	0.917
	education	1.117	0.597	1.872	0.082
Base + WML:SS	(Intercept)	64.106	10.014	6.402	<0.001
	lesion volume	0.066	0.094	0.704	0.493
	SS	0.055	0.635	0.086	0.933
	education	1.122	0.600	1.869	0.083
Base + WML:GCC	(Intercept)	63.961	9.057	7.062	<0.001
	lesion volume	0.073	0.039	1.893	0.079
	GCC	-0.590	5.286	-0.112	0.913
	education	1.117	0.597	1.872	0.082

Note. Lesion volume calculated at acute stage. SE = Standard Error; WML = white matter lesion; EC = external capsule; IFOF = inferior fronto-occipital fasciculus; SLF = superior longitudinal fasciculus; UF = uncinate fasciculus; BCC = body of the corpus callosum; SS = sagittal stratum; GCC = genu of the corpus callosum.

Table S6. Analysis of variance comparing full covariate + lesion models to base models for acute Word Prosody Recognition (affective prosody recognition) in RHS participants.

Model	Pseudo df	F	df	*p*-Value	FDR-Corrected *p*-Value
Base vs. WML:EC	22	79.56	4	<0.001	<0.001
Base vs. WML:IFOF	22	26.86	4	<0.001	0.0001
Base vs. WML:SLF	22	28.43	4	<0.001	0.0001
Base vs. WML:UF	22	45.43	4	<0.001	<0.001
Base vs. WML:BCC	22	16.94	4	0.0020	0.0020
Base vs. WML:SS	22	21.30	4	0.0003	0.0008
Base vs. WML:GCC	22	16.91	4	0.0020	0.0020

Note. RHS = right hemisphere stroke; df = degrees of freedom; FDR = false discovery rate; WML = white matter lesion; EC = external capsule; IFOF = inferior fronto-occipital fasciculus; SLF = superior longitudinal fasciculus; UF = uncinate fasciculus; BCC = body of the corpus callosum; SS = sagittal stratum; GCC = genu of the corpus callosum.

Table S7. Analysis of variance comparing full covariate + lesion models to base models for acute Recognition of Prosodic Features (Stage 1) task in RHS participants.

Model	Pseudo df	F	df	*p*-Value	FDR-Corrected *p*-Value
Base vs. WML:EC	23	0.38	2	0.8282	0.8282
Base vs. WML:IFOF	23	0.49	2	0.7830	0.8282
Base vs. WML:SLF	23	4.53	2	0.1040	0.3120
Base vs. WML:UF	23	12.21	2	0.0022	0.0134
Base vs. WML:BCC	23	10.19	2	0.0061	0.0245
Base vs. WML:SS	23	22.58	2	<0.001	0.0001
Base vs. WML:GCC	23	11.22	2	0.0037	0.0183

Note. RHS = right hemisphere stroke; df = degrees of freedom; FDR = false discovery rate; WML = white matter lesion; EC = external capsule; IFOF = inferior fronto-occipital fasciculus; SLF = superior longitudinal fasciculus; UF = uncinate fasciculus; BCC = body of the corpus callosum; SS = sagittal stratum; GCC = genu of the corpus callosum.

Table S8. Analysis of variance comparing full covariate + lesion models to base models for acute Matching Features to Emotion (Stage 2) task in RHS participants.

Model	Pseudo df	F	df	*p*-Value	FDR-Corrected *p*-Value
Base vs. WML:EC	21	24.66	4	0.0001	0.0003
Base vs. WML:IFOF	21	22.25	4	0.0002	0.0007
Base vs. WML:SLF	21	61.73	4	<0.001	<0.001
Base vs. WML:UF	21	28.09	4	<0.001	0.0001
Base vs. WML:BCC	21	19.40	4	0.0007	0.0013
Base vs. WML:SS	21	17.06	4	0.0019	0.0019
Base vs. WML:GCC	21	19.48	4	0.0006	0.0013

Note. RHS = right hemisphere stroke; df = degrees of freedom; FDR = false discovery rate; WML = white matter lesion; EC = external capsule; IFOF = inferior fronto-occipital fasciculus; SLF = superior longitudinal fasciculus; UF = uncinate fasciculus; BCC = body of the corpus callosum; SS = sagittal stratum; GCC = genu of the corpus callosum.

Table S9. Analysis of variance comparing full covariate + lesion models to base models for acute Emotion Synonym Matching (Stage 3) task in RHS participants.

Model	Pseudo df	F	df	*p*-Value	FDR-Corrected *p*-Value
Base vs. WML:EC	19	21.09	3	0.0001	0.0001
Base vs. WML:IFOF	19	23.55	3	<0.001	0.0001
Base vs. WML:SLF	19	40.09	3	<0.001	<0.001
Base vs. WML:UF	19	36.59	3	<0.001	<0.001
Base vs. WML:BCC	19	42.01	3	<0.001	<0.001
Base vs. WML:SS	19	24.10	3	<0.001	0.0001
Base vs. WML:GCC	19	43.88	3	<0.001	<0.001

Note. RHS = right hemisphere stroke; df = degrees of freedom; FDR = false discovery rate; WML = white matter lesion; EC = external capsule; IFOF = inferior fronto-occipital fasciculus; SLF = superior longitudinal fasciculus; UF = uncinate fasciculus; BCC = body of the corpus callosum; SS = sagittal stratum; GCC = genu of the corpus callosum.

Table S10. Analysis of variance comparing full covariate + lesion models to base models for acute Emotional Facial Expression Recognition (domain-general emotion) task in RHS participants.

Model	Pseudo df	F	df	*p*-Value	FDR-Corrected *p*-Value
Base vs. WML:EC	17	16.03	3	0.0011	0.0033
Base vs. WML:IFOF	17	25.84	3	<0.001	0.0001
Base vs. WML:SLF	17	14.76	3	0.0020	0.0041
Base vs. WML:UF	17	10.39	3	0.0155	0.0155
Base vs. WML:BCC	17	19.16	3	0.0003	0.0013
Base vs. WML:SS	17	17.25	3	0.0006	0.0025
Base vs. WML:GCC	17	19.55	3	0.0002	0.0013

Note. RHS = right hemisphere stroke; df = degrees of freedom; FDR = false discovery rate; WML = white matter lesion; EC = external capsule; IFOF = inferior fronto-occipital fasciculus; SLF = superior longitudinal fasciculus; UF = uncinate fasciculus; BCC = body of the corpus callosum; SS = sagittal stratum; GCC = genu of the corpus callosum.
